# ICA/SDF‐1α/PBMSCs loaded onto alginate and gelatin cross‐linked scaffolds promote damaged cartilage repair

**DOI:** 10.1111/jcmm.18236

**Published:** 2024-03-20

**Authors:** Pengzhen Wang, Pingping Zhu, Wenhui Yin, Jian Wu, Shaoheng Zhang

**Affiliations:** ^1^ Guangzhou Institute of Traumatic Surgery Guangzhou Red Cross Hospital of Jinan University Guangzhou Guangdong China; ^2^ Key Laboratory of Regenerative Medicine, Ministry of Education Jinan University Guangzhou Guangdong China; ^3^ Department of Neurology Guangzhou Red Cross Hospital of Jinan University Guangzhou Guangdong China; ^4^ Department of Cardiology Guangzhou Red Cross Hospital of Jinan University Guangzhou Guangdong China; ^5^ Department of Otorhinolaryngology Guangzhou Red Cross Hospital of Jinan University Guangzhou Guangdong China

**Keywords:** alginate, cartilage, gelatin, icariin, SDF‐1α

## Abstract

A three‐dimensional alginate‐coated scaffold (GAIS) was constructed in the present study to showcase the multidifferentiation potential of peripheral blood mesenchymal stem cells (PBMSCs) and to investigate the role and mechanism by which Icariin (ICA)/stromal cell‐derived factor (SDF‐1α)/PBMSCs promote damaged articular repair. In addition, the ability of ICA, in combination with SDF‐1α, to promote the migration and proliferation of stem cells was validated through the utilization of CCK‐8 and migration experiments. The combination of ICA and SDF‐1α inhibited the differentiation of PBMSCs into cartilage, as demonstrated by in vivo experiments and histological staining. Both PCR and western blot experiments showed that GAIS could upregulate the expression of particular genes in chondrocytes. In comparison to scaffolds devoid of alginate (G0), PBMSCs seeded into GAIS scaffolds exhibited a greater rate of proliferation, and the conditioned medium derived from scaffolds containing SDF‐1α enhanced the capacity for cell migration. Moreover, after a 12‐week treatment period, GAIS, when successfully transplanted into osteochondral defects of mice, was found to promote cartilage regeneration and repair. The findings, therefore, demonstrate that GAIS enhanced the in vitro capabilities of PBMSCs, including proliferation, migration, homing and chondrogenic differentiation. In addition, ICA and SDF‐1α effectively collaborated to support cartilage formation in vivo. Thus, the ICA/SDF‐1α/PBMSC‐loaded biodegradable alginate‐gelatin scaffolds showcase considerable potential for use in cartilage repair.

## 1 INTRODUCTION

The inherent properties of articular cartilage, including its non‐neurological, avascular and low cell density characteristics, restrict its ability to effectively respond to inflammation and damage.^1^, ^2^ In addition, the long‐term wear and abrupt tearing of articular cartilage contribute to the progression of cartilage damage and the development of osteoarthritis (OA). OA or cartilage damage significantly affects the daily life and work of individuals afflicted with joint swelling and stiffness.^3^ According to preliminary calculations, it is projected that by 2025, the well‐being and overall quality of life for a population of approximately 300 million individuals worldwide will be affected.^4^ Currently, traditional medical treatment and surgical interventions comprise the primary approaches employed for addressing cartilage injury.^5^ These methods offer certain benefits in impeding the advancement of cartilage damage or OA. However, they do not possess any advantages in preventing cartilage deterioration or achieving complete healing of cartilage trauma.^5^

Traditional surgical procedures, such as autografts, microfractures and allografts, face limitations in their practical applications due to factors such as a scarcity of donors and inadequate production of hyaline cartilage.^6^ Consequently, the investigation of synthetic cartilage has emerged as a prominent area of research. Not only can synthetic cartilages be manufactured on a significant scale, they can also be employed in clinical settings while ensuring biological safety. In recent years, there has been substantial progress in advancing 3D printing technology, particularly in its application for bone defect repair.^7^ As an emerging technology, 3D printing possesses the capability to fabricate scaffolds that precisely conform to the contours of bone defects.^8^ Furthermore, the internal porosity of these scaffolds also facilitates the accommodation of newly forming tissue, thereby promoting the development of blood vessels and bone tissue.^8^ Thus, the investigation of appropriate materials for tissue engineering scaffolds holds significant importance.^9^ Among the range of biological materials developed for tissue engineering applications, gelatin and alginate bioactive glass are characterized by their commendable biocompatibility, appropriate degradability and superior osteoinductive properties.^10^ As a result, they are considered to be highly suitable for use in bone defect repair.

Due to its natural composition, alginate possesses inherent advantages in the fabrication of porous composites. Due to its biodegradability, absorption capabilities, adjustable mechanical properties and low pro‐inflammatory response, alginate has been widely used as a natural polymer. However, due to its resistance to shaping, alginate is particularly suitable for cross‐linking with additional scaffold materials in order to fabricate hydrogels intended for biological applications.^11^ In this context, cell tissue engineering has made exclusive use of 3D porous gelatin scaffolds due to their exceptional biocompatibility and high porosity.^12^ However, the continuous repair of cartilage defects using gelatin presents numerous challenges owing to its susceptibility to degradation and inadequate mechanical strength. In this context, the combination of gelatin and alginate has been demonstrated to fulfil the requirements of cartilage repair.^13^

An ideal cartilage repair scaffold should possess the capability to load and continuously release growth factors and drugs, as well as recruit a sufficient number of mesenchymal stem cells (MSCs) from the subchondral bone to undergo differentiation into cartilage.^14^ The use of traditional pharmaceutical steroids for cartilage repair has been observed to elicit greater side effects.^15^ Thus, while transplanting chondrocytes directly from individual tissues has been observed to yield significant results, the use of pharmaceutical steroids reduces the capacity for chondrocytes to differentiate when subjected to excessive monolayer culture and harvesting.^16^ In this context, the engineering of cartilage and bone tissue has surfaced as a highly promising approach that requires minimally invasive procedures. Accordingly, the use of these tissue engineering strategies has been found to gradually restore cartilage to its initial stage by stimulating the repair capacity of PBMSCs via use of suitable scaffolds, effective drugs and protein factors.^17^ In this context, the microenvironment created by porous biological scaffolds to facilitate seed cell differentiation, growth and secretion of cellular components is deemed highly essential for cartilage regeneration.^18^

As a familiar chemokine, SDF‐1α plays a crucial role in facilitating the migration of stem cells towards the site of injury in order to repair tissue via the SDF‐1‐CXCR4 axis.^19^ Given that endogenous stem cells are the primary repairing seed cells, it is critical in cartilage engineering to establish an optimal microenvironment for the differentiation of endogenous stem cells into cartilage in the defect site. Previous approaches involved preferential selection for MSCs derived from bone marrow; however, obtaining bone marrow is difficult and employs a traumatic approach.^20^ Earlier studies have confirmed that peripheral blood‐MSCs also possess the characteristics of stem cells.^21^ Furthermore, due to the accessibility, sufficiency and non‐invasive nature of peripheral blood, peripheral blood MSCs exhibit great potential for use in cell engineering applications.

Icariin (ICA), derived from the desiccated stems and leaves of *Epimedium* spp., exhibits profound anti‐inflammatory, anti‐tumour, anti‐oxidant and bone‐protective effects.^22^, ^23^ In addition, the use of ICA has also been observed to promote the differentiation of bone marrow MSCs and cartilage progenitor cells into chondrocytes in rat OA models.^24^ Because of its ability to promote the differentiation of MSCs, ICA has the ability to resolve the low chondrogenic differentiation of MSCs from autologous tissues.^24^ In addition, ICA places a high emphasis on maintaining an environment that promotes differentiation and inhibits inflammation. Furthermore, ICA and SDF‐1α are readily diffused in body tissues. Therefore, maintaining ICA and SDF‐1α at a certain concentration in the damaged area is critical. In the present study, a cross‐linking reaction was used to fabricate an alginate‐coated gelatin scaffold that simultaneously contained ICA and SDF‐1α. The biological properties of the scaffold complex were assessed through in vitro experiments, while the therapeutic effects of scaffold materials on a mouse model with osteochondral defects were investigated in vivo. The findings of this study suggest that the application of an alginate‐gelatin complex containing ICA and SDF‐1α demonstrates promising efficacy in the repair and regeneration of damaged cartilage.

## 2 RESULTS

### 2.1 Characterization of composite scaffolds

**FIGURE 1:**
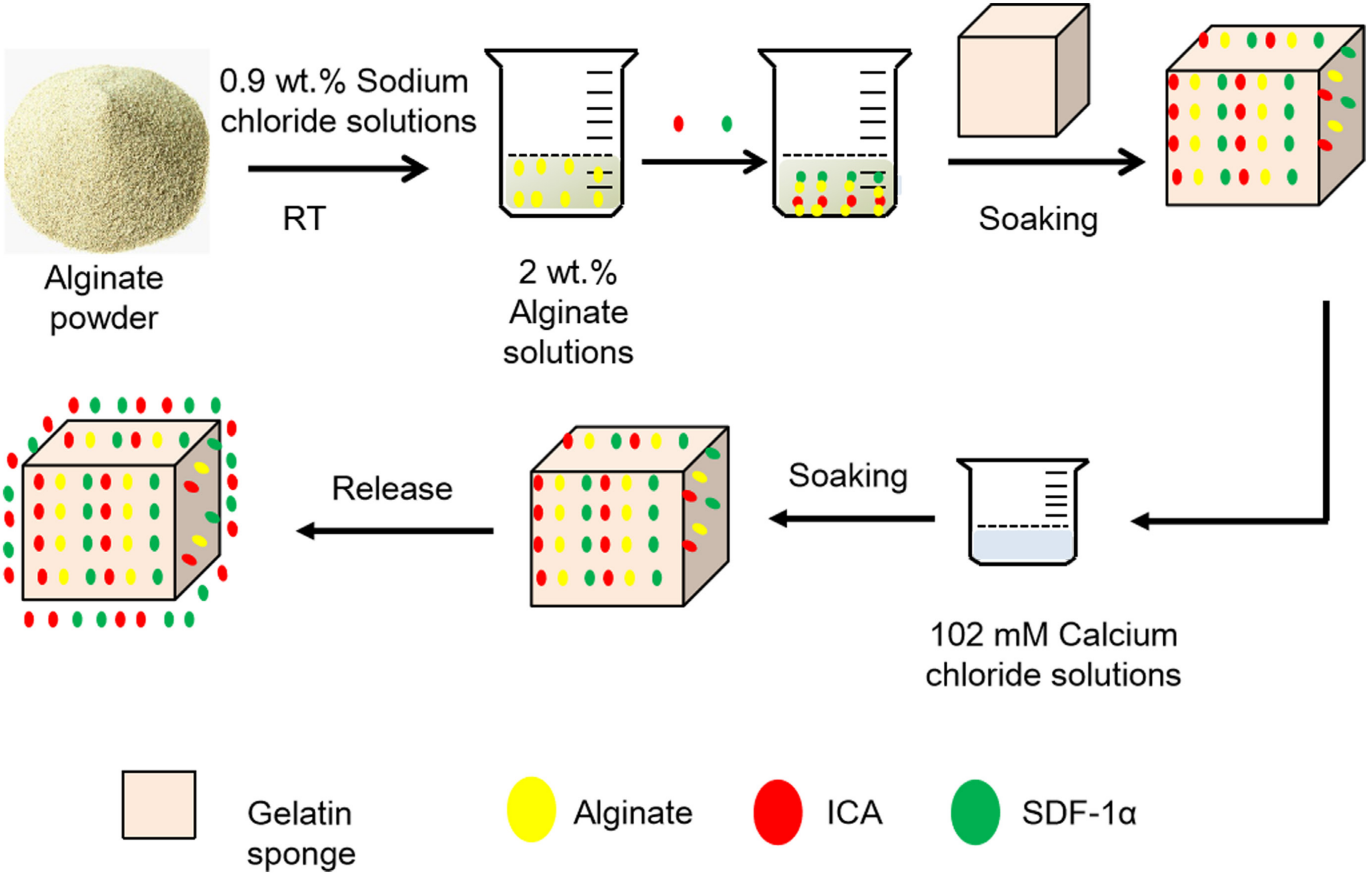
Simple flow chart of cross‐linked complexes containing alginate, ICA, and SDF‐1α.

**FIGURE 2:**
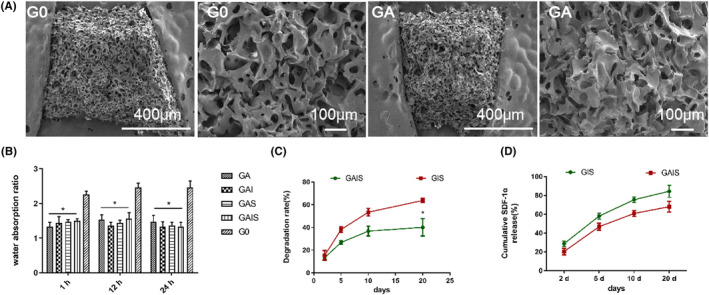
Conventional characteristics of composite stents. (A) SEM picture of alginate crosslinked and uncrosslinked scaffolds; (B) The water absorption performance; degradation performance (C) of the five groups of stents; and the slow release performance of SDF‐1α of the GIS and GAIS stents into the culture medium (D). *p < 0.05, compared with GA (B) or GIS (C, D).

The alginate‐ICA/SDF‐1α‐gelatin scaffold was formulated in accordance with the schematic diagram illustrated in Figure 1. Although the G0 and GA stents had a comparable morphology (Figure 2A), the structure of GA (diameter: 50–100 μm) was more densely packed than the G0 (diameter: 100–200 μm). Additionally, the water absorption rate of all alginate‐coated stents (GA, GAI, GAS and GAIS) was significantly lower than G0, as illustrated in Figure 2B. However, the as‐observed difference in water absorption between the alginate‐coated stents (GA, GAI, GAS and GAIS) was negligible. Moreover, GAIS (35%) exhibited a reduced degradation rate compared to GIS (65%) throughout the testing period (Figure 2C). Additionally, the ELISA test revealed that the release of SDF‐1α was comparatively slower in GAIS than in GIS. As a result, GIS released 84% SDF‐1α after 21 days, whereas GAIS released 70% SDF‐1α (Figure 2D).

### 2.2 Characteristics of PBMSCs

**FIGURE 3:**
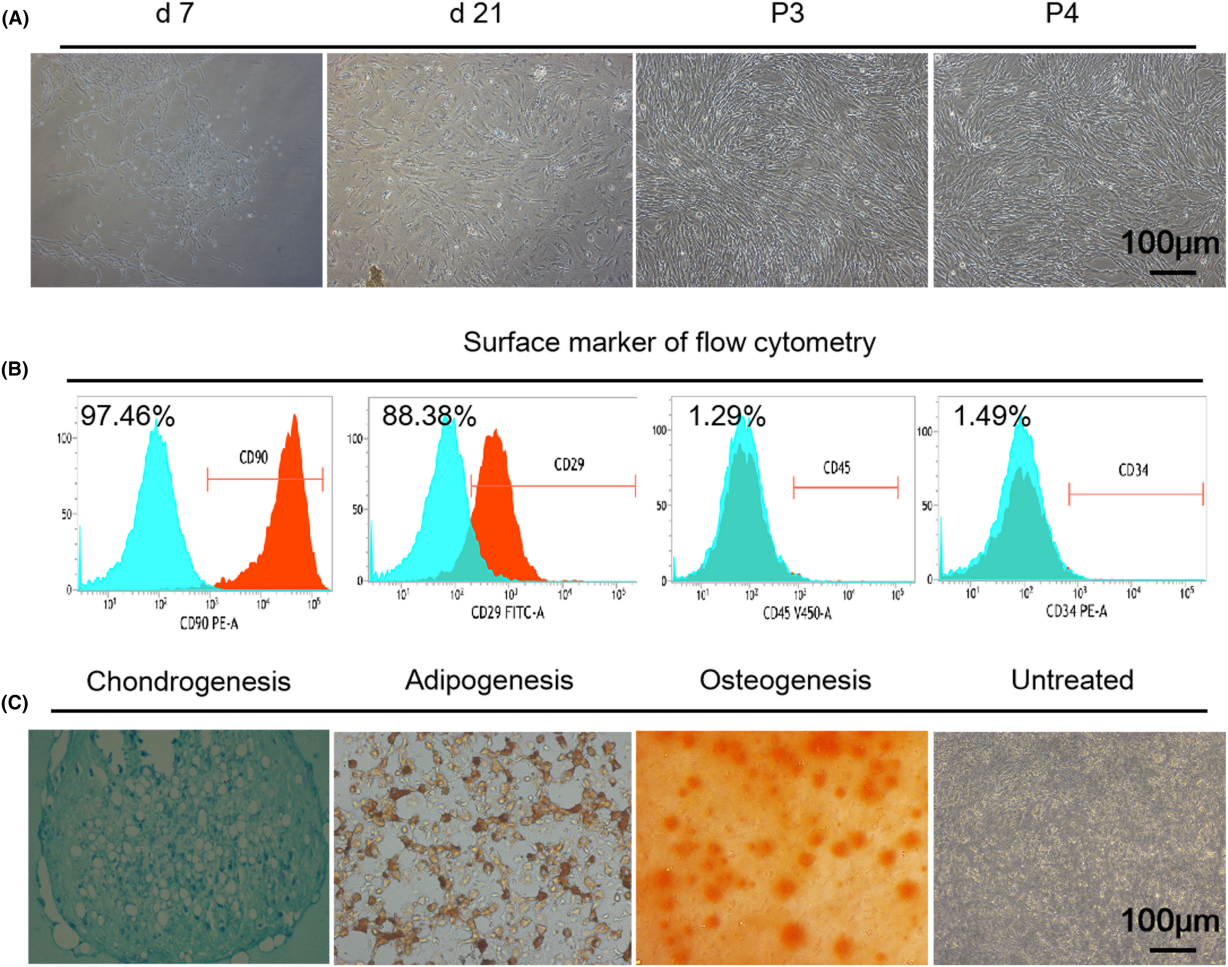
Characteristics of peripheral blood mesenchymal stem cells. (A) The cell morphology of PBMSCs at 7 days, 21 days after inoculation, and the third passage (P3) and fourth passage (P4) PBMSCs. (B) Flow cytometric identification of PBMSC surface markers. (C) Chondrogenic induction, lipid droplet induction and osteogenic induction of PBMSCs, 200×, scale: 100 μm.

Within the first 7 days, adherent PBMSCs assumed a spindle shape. Twenty‐one days later, after the initial 7 days, polygonal or fibroid cells completely covered the bottom of the culture flask; 14 days later, cell colonies formed. Appearing uniformly homogeneous and exhibiting a fibroblast‐like morphology (Figure 3A), adherent PBMSCs from the third passage (P3) and the fourth passage (P4) were utilized in subsequent experiments. As shown in Figure 3B, P4 PBMSC strongly expressed mesenchymal mass spectrum markers CD90 (97.46%) and CD29 (88.38%), whereas haematopoietic lineage markers CD34 (1.49%) and CD45 (1.29%) were barely expressed. Subsequently, PBMSCs were induced by adipogenic conditions to present cytoplasmic lipid droplets. The alcian blue staining test confirmed the presence of aggrecan in the chondrogenesis‐inducing cells. Furthermore, the use of Alizarin red staining revealed the presence of alizarin nodular aggregates at the base of the petri dish containing the osteogenic medium after a culture period of 21 days; the negative staining results for the multiple differentiation potential of PBMSCs is shown in Figure 3C.

### 2.3 Scaffold characteristics

**FIGURE 4:**
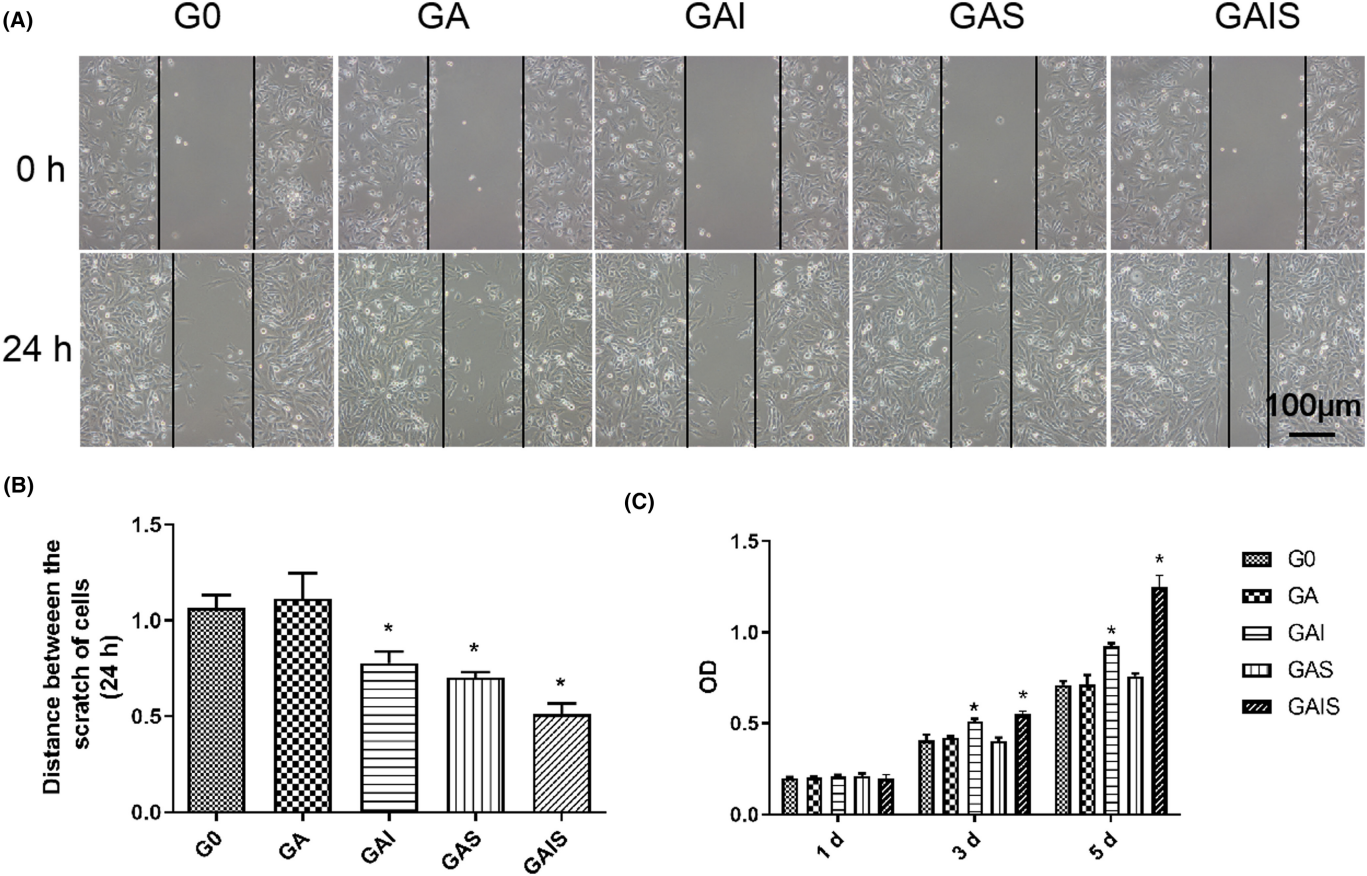
Migration and vitality assay. (A and B) The distance of the scratch edge was quantitatively analysed. (C) The proliferation assay. *p < 0.05, compared with G0 (A, B) or GA (C), 200×, scale: 100 μm.

PBMSCs cultured in conditioned medium from the GAS and GAIS groups migrated significantly faster than those cultured in conditioned medium from the G0, GA and GAI groups (Figure 4A,B). However, the difference between the growth rates of these cells and those from the GAS and GAIS group was not statistically significant. As shown in Figure 4C, the absorbance of PBMSCs on all scaffolds on Day 1 was virtually identical. Moreover, in contrast to G0 stents, both GAI and GAIS scaffolds notably facilitated the proliferation of absorbance in PBMSCs cultured for 3 and 5 days, respectively. Furthermore, as evident from the results of the cell viability test, both GAI and GAIS were observed to promote the proliferation of PBMSCs on the scaffold; however, the impact of GAIS on cell viability was more pronounced in comparison to GAI.

### 2.4 Histological characteristics of different scaffolds seeded with PBMSCs

**FIGURE 5:**
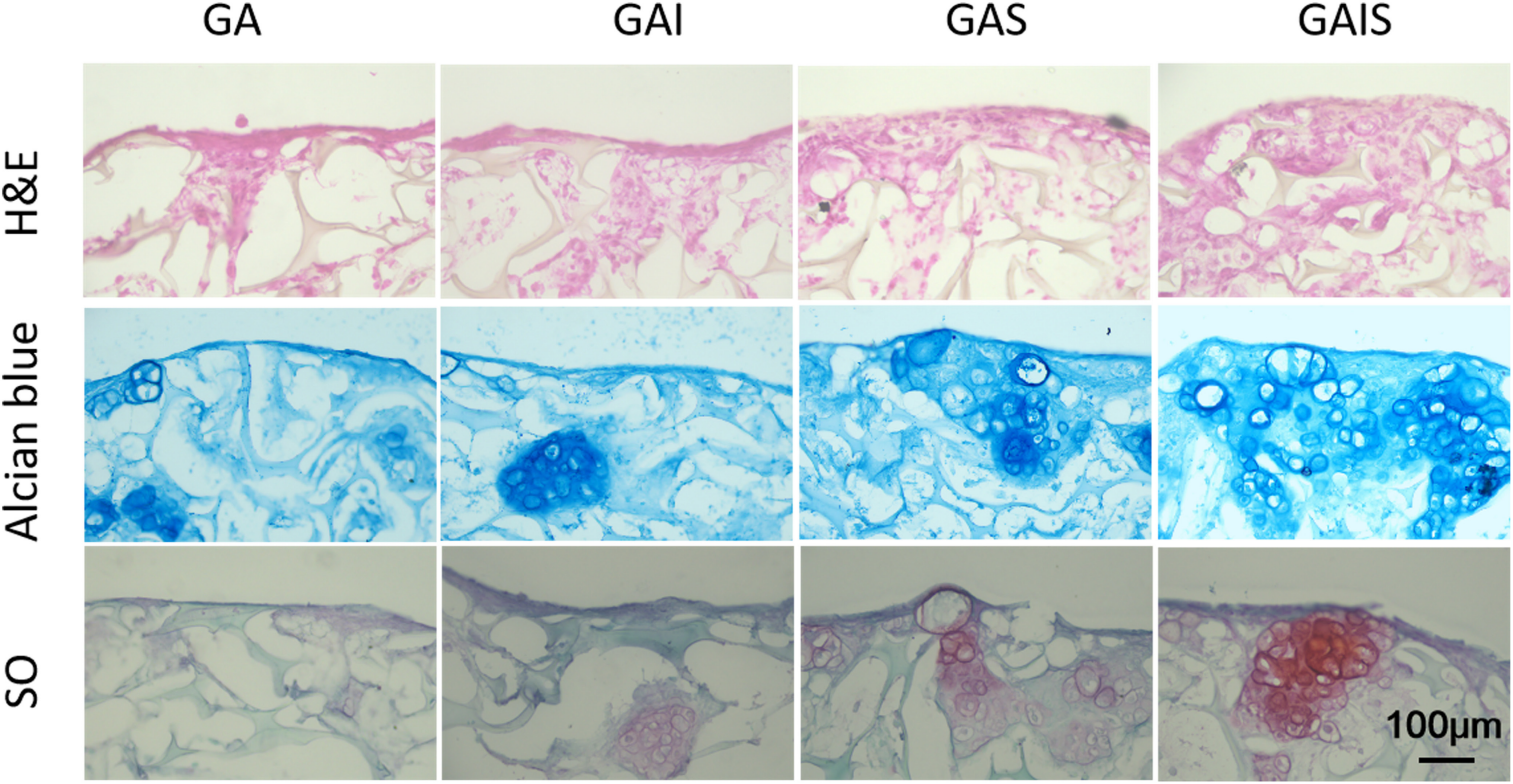
Histological images of PBMSCs cultured on scaffolds for 21 days. GA group was as the control. GA means that PBMSCs were cultured on gelatin‐alginate cross‐linked scaffolds with no treatment, GAI means that PBMSCs were cultured on gelatin‐alginate cross‐linked scaffolds treated with ICA, GAS means that PBMSCs were cultured on gelatin‐alginate cross‐linked scaffolds treated with SDF‐1α, GAIS means that PBMSCs were cultured on gelatin‐alginate cross‐linked scaffolds treated with ICA and SDF‐1α. 200×, scale: 100 μm.

To gain insight into the histological characteristics of different scaffolds seeded with PBMSCs, chondrogenic differentiation and cartilaginous ECM formation were first assessed using frozen section and morphological staining. The haematoxylin and eeosin (H&E) staining revealed that GAI, GAS and GSTS contained a greater number of cells than GA (Figure 5). Furthermore, the combination of ICA and SDF‐1α enhanced cell adhesion and proliferation. In addition, the presence of alcian blue indicated that the PBMSCs had successfully differentiated into chondrocytes. However, the staining area of alcian blue and (safranin O‐fast green) SO was considerably smaller in the GA group compared to the GAI, GAS and GAIS groups. Notably, the largest area of alcian blue and SO staining was observed in the GAIS group. Thus, the findings suggest that ICA and SDF‐1α have the capacity to stimulate PBMSCs to differentiate into chondrocytes seeded on alginate‐coated scaffolds. Notably, the combined impact of ICA and SDF‐1α on PBMSCs was particularly evident.

### 2.5 Expressions of genes and protein related to chondrogenic differentiation and cartilage degeneration in PBMSCs

**FIGURE 6:**
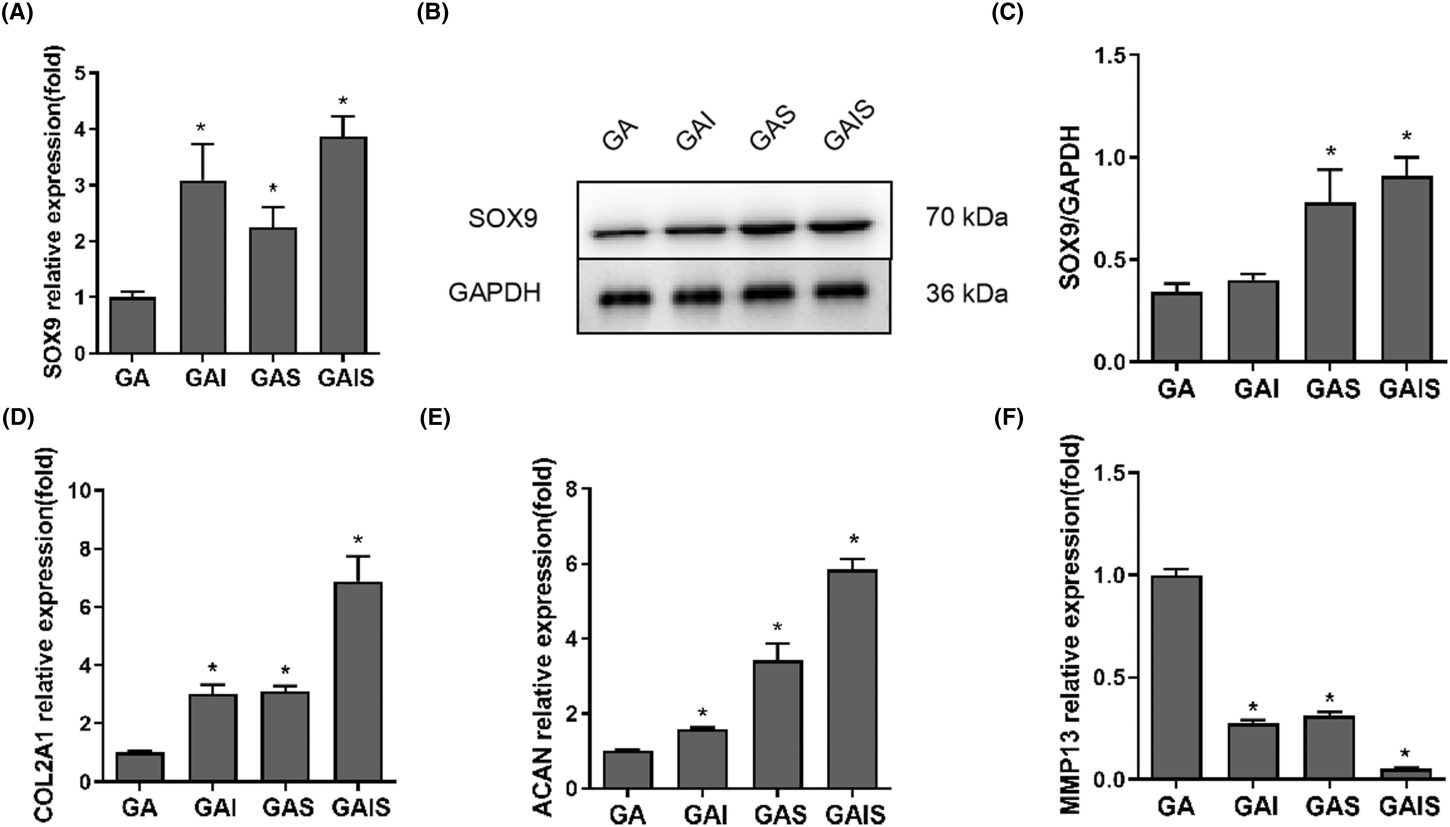
Expressions of genes and proteins related to chondrogenic differentiation and cartilage degeneration in PBMSCs cultured on scaffolds for 21 days. (A) mRNA expression of SOX9. (B and C) Protein expression and quantitative analysis of SOX9. (D–F) mRNA expression of COL2A1, ACAN, and MMP13. *p < 0.05, compared with GA.

As shown in Figure 6, ICA/SDF‐1α scaffolds enhanced the chondrogenic differentiation capability of PBMSCs. Furthermore, in contrast to GA, both GAI, GAS, as well as GAIS significantly inhibited MMP13 mRNA expression while significantly promoting the expression of SOX9, COL2A1 and ACAN mRNA. Moreover, the bands obtained from the western blot assay demonstrated that GAI, GAS and GAIS significantly increased the expression of the SOX9 protein in PBMSCs when compared to GA. Thus, the scaffold containing ICA/SDF‐1α demonstrated a significant potential to facilitate the conversion of PBMSCs into cartilage.

### 2.6 Evaluation of cartilage repair level in vivo

**FIGURE 7:**
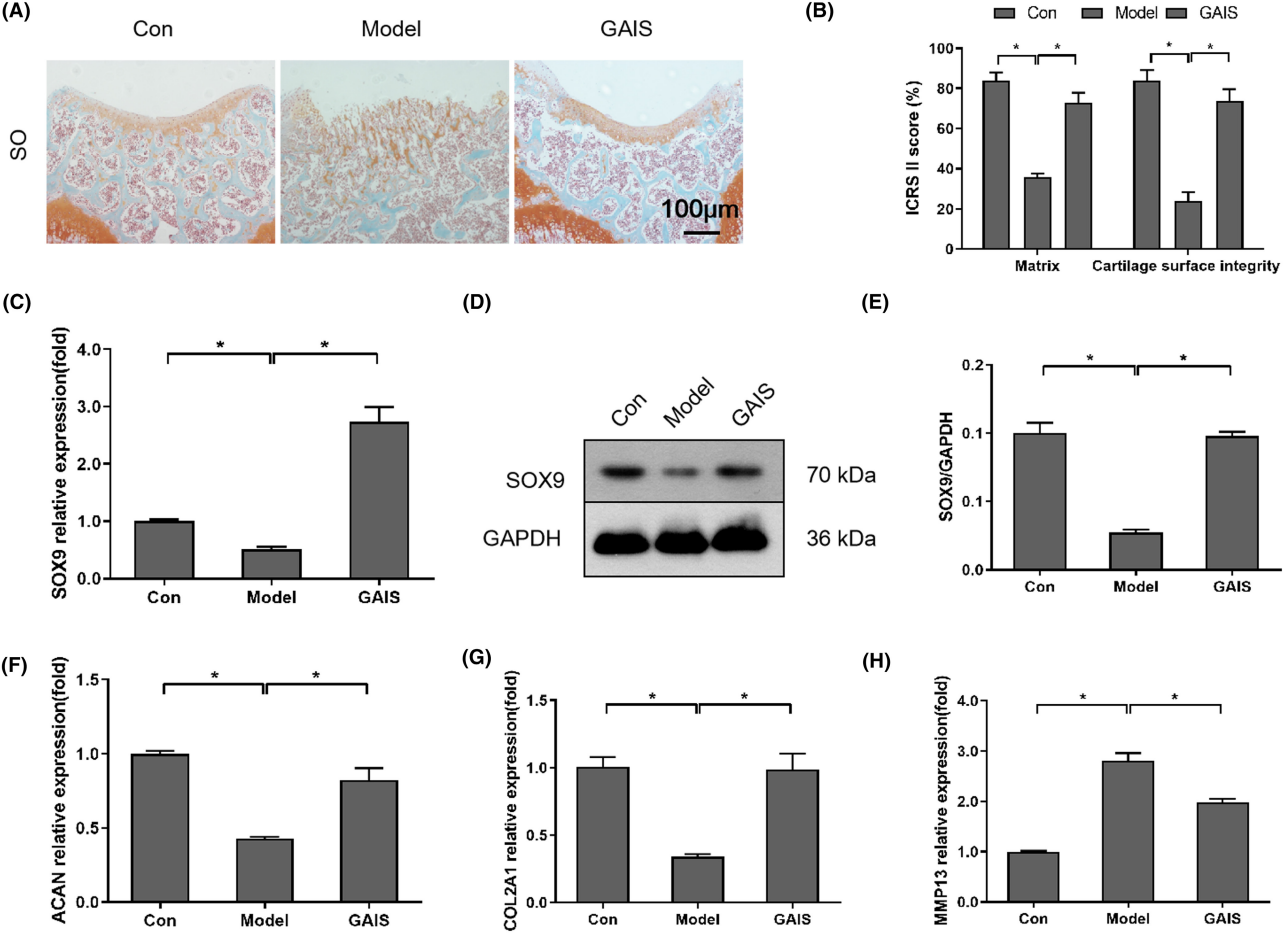
GAIS reduced cartilage degradation and increased SOX9 expression in osteochondral the defect model of mice. (A) SO staining of mice 12 weeks after surgery. (B) ICRS II score for cartilage repair degree of all groups. (C–E) SOX9 expression in mice 12 weeks after surgery. (F–H) The expressions of ACAN, COL2A1 and MMP13 mRNA in mice 12 weeks after surgery. *p < 0.05, 200×, scale: 100 μm.

As illustrated in Figure 7A,B, osteochondral defect surgery resulted in a reduction in the extent of SO staining; in contrast, GAIS treatment restored the reduced SO staining. Furthermore, in terms of matrix staining and articular surface integrity, the model group obtained a significantly lower ICRS II score than the control group. Conversely, ICRS II score was significantly improved by GAIS treatment but diminished by osteochondral defect surgery.

In addition, SOX9 expression was significantly higher in the GAIS groups compared to the model and control groups, as shown by WB and qPCR results (Figures 7C–E). Furthermore, when compared to the model group, the levels of COL2A1 and ACAN qPCR mRNA expression were significantly upregulated in the GAIS group (Figures 7F–H). Moreover, when compared to the model group, the qPCR results for MMP13 in the GAIS group were drastically different (Figures 7F–H). Thus, the findings of this study suggest that the alginate‐gelatin complex containing ICA and SDF‐1α could effectively promote the repair and regeneration of damaged cartilage.

## 3 DISSCUSSION

In the dynamic domain of clinical and basic medicine, an increasing number of researchers are endeavouring to identify 3D stents that exhibit favourable absorbability and compatibility for the purpose of cartilage repair.^25^, ^26^, ^27^ In this context, it is ideal to incorporate suitable drugs onto the scaffold intended for repairing cartilage damage; such drugs may have the potential to stimulate the in situ differentiation of stem cells into cartilage.^28^, ^29^ The present study utilized a compound composed of alginate, gelatin and two drugs, ICA and SDF‐1α, to induce stem cell migration and differentiation. In‐depth studies have demonstrated that the complex not only facilitates the development of cartilage in PBMSCs cultured in vitro under 3D conditions but also stimulates the regeneration and repair of subchondral bone damage in mice.

Alginate‐based scaffolds are often characterized by their ability to demonstrate favourable biocompatibility, a reduced degradation rate, and the ability to release substances gradually.^30^ In the present study, we assembled and adapted an alginate cross‐linked gelatin scaffold possessing a degradation rate suitable for our purposes. Additionally, we incorporated two active drugs (ICA and SDF‐1α) into the scaffold to facilitate the process of cartilage regeneration. As evident from earlier studies, the incorporation of alginate into the scaffold has been found to have a notable impact on reducing both water absorption and degradation rate.^31^ Thus, the present study demonstrates that the use of an alginate‐coated gelatin scaffold, possessing an appropriate degradation rate, has the potential to enhance the process of cartilage formation within the designated timeframe for repair.

Earlier studies have indicated that the cellular outcomes, including cell survival, differentiation and intracellular component synthesis, during the process of cartilage remodelling and repair are influenced by the 3‐dimensional structure and pore size of the material.^32^ Accordingly, a scaffold with a pore size ranging from100 to 300 μm demonstrates optimal suitability for promoting cell survival, migration and homing.^33^ In the present study, the viability and proliferation ability of the PBMSCs that were seeded on the scaffold with a network structure were found to be higher compared to the control group. Furthermore, earlier studies have also documented that collagen scaffolds sourced from animal skin and hair, featuring a pore size of 100–300 μm exhibit an enhanced efficacy in preserving and promoting the cartilage phenotype.^34^ In this context, the alginate‐coated gelatin stent utilized in this study was considered to exert a significant impact on the process of cartilage repair.

To facilitate the repair of the injured area, the biologically active factor was encased within the materials implanted from the injured region in the present study. In this context, the alginate‐coated gelatin was selected in this study on account of its favourable absorption capacity and slow‐release property.^35^ Earlier studies demonstrate that ICA could be entrapped and coated using hydroxyapatite/alginate (HAA) porous scaffolds.^36^ In addition to facilitating stem cell migration and homing, scaffolds containing ICA and SDF‐1α sustain a microenvironment loaded with chondrogenic growth factors.^37^, ^38^ In this context, SDF‐1α facilitates the engraftment, retention, migration and homing of stem cells in bone marrow.^39^, ^40^ In the present study, SDF‐1α was observed to promote the migration and homing of PBMSCs, which is consistent with findings reported in the literature.^41^

Previous studies have also demonstrated the capacity of ICA to enhance the generation of cartilage by stem cells when used in conjunction with materials like polyhydrogels or magnetic nanocapsules.^42^ Currently however, there is a lack of research regarding the adsorption of the active monomer ICA in conjunction with the growth factor SDF on the scaffold composed of alginate cross‐linked gelatin. The findings of the research study demonstrated that the ICA/SDF‐1α alginate gelatin scaffold facilitated the recruitment of stem cells to the designated site and facilitated the differentiation of PBMSCs into cartilage, thereby effectively facilitating the repair of cartilage damage.

While the present study demonstrated the capacity of GAIS to prompt the differentiation of stem cells into chondrocytes both in vitro and in vivo, the underlying mechanism was not thoroughly elucidated in this study. In this context, further investigation is warranted to thoroughly examine the capacity of GAIS to prompt the differentiation of stem cells in subsequent studies. Thus, to conclude, this article has successfully arrived at a robust conclusion that the utilization of an alginate scaffold incorporating ICA and SDF‐1α effectively facilitates the differentiation of stem cells into chondrocytes and promotes cartilage regeneration. This finding, therefore, establishes a theoretical foundation and offers future research avenues for the therapeutic intervention of cartilage injuries.

## 4 MATERIALS AND METHODS

### 4.1 Construction of alginate‐coated gelatin porous composites

A solution of alginate was prepared by dissolving 2% (w/v) of alginate in a solution of 0.9% sodium chloride at room temperature. The alginate solutions were filtered using a filter membrane and subsequently stored at a temperature of 4°C. The prepared solution containing a mixture of ICA and SDF‐1α in alginate solution had a concentration of 10^−6^ M and 300 ng/mL.^20^ The gelatin samples were purchased from Jinling Pharmaceutical Co., Ltd. The sterile gelatin scaffolds were sectioned into cubes (measuring 2 × 2 × 2 mm^3^) using sterilized scissors. Subsequently, the scaffolds were exposed to ultraviolet light for a duration of approximately 5 h. The gelatin was submerged in sterilized calcium chloride (CaCl_2_) solutions in order to create a cross‐linked complex. This resulted in the formation of gelatin‐alginate scaffolds that were loaded with ICA and SDF‐1α. The designation ‘G0’ refers to scaffolds that do not contain alginate; ‘GA’ denotes scaffolds that solely consist of alginate; ‘GAI’ denotes scaffolds that contain both alginate and ICA. ‘GAS’ represents scaffolds that contain both alginate and SDF‐1α; and ‘GAIS’ signifies scaffolds that contain alginate, ICA and SDF‐1α simultaneously. In addition, GIS refers to the scaffolds that incorporate both ICA and SDF‐1α.

### 4.2 Characterization of scaffolds

#### 4.2.1 Scanning for pore size of GA and G0 by electron microscope

The microscopic morphology of GA and G0 was assessed using a scanning electron microscope (SEM, model CX‐200 TA, ROK; voltage: 5.0 kV). The measurement of pore size in GA and G0 was conducted using Image‐Pro Plus 6.0 software.

#### 4.2.2 Water absorption capacity analysis

The gelatin sample (measuring 2 × 2 × 2 mm^3^), was promptly immersed in 2 mL of phosphate‐buffered saline (PBS) at a temperature of 37°C prior to conducting a dry weighing procedure (W0). The wet weight (W1) was measured at three time points: 1, 12 and 24 h using the equation shown below:

(1) $$\left[\text{Water absorption ratio}\;\left(\%\right)=\frac{W1-W0}{W0}\right].$$

The equation was employed to determine the water absorption capacity. Each gelatin stent underwent a minimum of three testing trials.

#### 4.2.3 Analysis of degradation ability and drug release potential of scaffolds

The initial measurement of W2 involved determining the wet weight of GAIS and GIS samples that were incubated in 2 mL of PBS at a temperature of 37°C. The weight of the scaffolds treated with 2 mL of PBS was measured and recorded on Day 2, 5, 10 and 20 using the equation depicted below:

(2) $$\left[\text{Water absorption ratio}\;\left(\%\right)=\frac{W2-W3}{W2}\right].$$

The equation was employed to assess the degradability of the scaffold. PBS was collected on Day 2, 5, 10 and 20, in a sequential manner. Subsequently, the cumulative concentration of SDF‐1α released was determined through the utilization of enzyme‐linked immunosorbent assay (ELISA). Each gelatin stent was tested for a minimum of three times.

#### 4.2.4 Isolation and culture of PBMSCs

The present investigation was conducted at the Institute of Trauma Surgery in Guangzhou and received approval from the Ethics Committee of Guangzhou Red Cross Hospital (No. 2021‐033‐02). Based on the existing literature,^12^ a wound measuring 1 cm × 1 cm was intentionally created on the dorsal region of the mice. The wound was subjected to daily disinfection, while the mice were provided unrestricted access to water to facilitate their normal physiological functions. After a period of 1 week, the mice were subjected to anaesthesia using a 0.8% pentobarbital solution administered via the abdominal cavity. A total of 3 mL of blood from the abdominal aorta was obtained from a cohort of 10 mice using a fine needle that was preloaded with heparin at a concentration of 300 U/mL. The collected sample was subsequently mixed with an equal volume of PBS and monocytes were isolated by centrifuging the sample at 2000 g using Ficoll separation solution (17144002, GE, USA). The mononuclear cells (MNCs) obtained from the middle layer solutions were collected and placed in T‐25 culture flasks at a concentration of 2 × 10^5^/mL. The culture medium used was DMEM supplemented with 1% penicillin/streptomycin, 20% fetal bovine serum (FBS) and 20 ng/mL basic fibroblast growth factor (bFGF). The cell culture environment was maintained at a stable temperature of 37°C, while the incubator was maintained with a CO_2_ concentration of 5%. After being cultured for a duration of approximately 21 days, it was observed that the cells occupied approximately 80% of the total surface area at the bottom of the flask. The cells from the fourth passage were utilized for subsequent in vitro experiments, which involved the identification of surface markers for stem cells and pluripotency differentiation.

#### 4.2.5 PBMSC phenotypic identification

A total of 1 × 10^6^ PBMSCs were introduced into a 1 mL PBS solution contained within a tube. A volume of 100 μL of prepared solutions containing anti‐mouse CD90 (1:100; ab225; abcam; Massachusetts, USA), anti‐mouse CD29 (1:100; ab179472; abcam), anti‐mouse CD34 (1:100; ab81289; abcam) and anti‐mouse CD45 (1:100; ab10558; abcam) were added to tubes containing 1 mL of cell suspension in order to facilitate antigen binding. The PBMSC phenotype was then analysed using a flow cytometer (FACSCalibur, BD) equipped with Cell Quest analytic software.

#### 4.2.6 Adipogenic, osteogenic and chondrogenic differentiation

In the context of osteogenesis, the P4 PBMSCs (3 × 10^4^/well) were introduced into 6‐well plates and subjected to osteogenic differentiation using a commercially available osteogenic differentiation medium kit (MUXMX‐90021, OriCell®, Cyagen, Soochow, China). The cells were then cultured for a duration of 14 days, and the evaluation of osteogenesis was performed through alizarin red staining, following a previously established protocol.^20^ To initiate chondrogenesis, a total of 3 × 10^5^ PBMSCs were placed in a centrifuge tube and subjected to centrifugation at a force of 2000 g. This process facilitated the formation of micelles by the cells. Subsequently, chondrocyte mass was induced for a duration of 21 days using a commercially available chondrogenic differentiation medium kit (MUXMX‐90041, Cyagen). The resulting differentiation was assessed through alcian blue staining, a method commonly employed for the identification of differentiation.^26^ To initiate adipogenesis, P4 PBMSCs were seeded at a density of 1 × 10^5^ cells per well in a 6‐well plate. The cells were then cultured for a period of 21 days using a commercially available adipogenic differentiation medium kit (MUXMX‐90031, Cyagen). Oil red staining was conducted following a 3‐week period of induction.^20^

#### 4.2.7 Cell migration

Conditioned media was collected from various scaffold groups, including G0, GA, GAI, GAS and GAIS, by individually soaking these cubic scaffolds in DMEM containing 1% FBS for a duration of 24 h. Subsequently, PBMSCs (1 × 10^5^ cells/well) were seeded in a mini‐well plate. A straight line was drawn in the center of the well using a sterile 200 L pipette tip, once the PBMSCs had nearly completely covered the bottom of the petri dish. Following two washes with PBS, PBMSCs were subjected to an additional 24 h of treatment with the scaffold‐conditioned medium that was collected. An inverted phase‐contrast microscope (Ti‐U; Nikon Corporation) was then utilized to capture images of the scratches. The migration ability of PBMSCs in each group was assessed by calculating the width of the two edges using ImageJ software.

#### 4.2.8 CCK‐8 assay

A CCK‐8 kit (Dojindo, Japan) was utilized to assess the viability of PBMSCs seeded onto the scaffolds at a density of 1 × 10^5^/scaffold. Following culturing for 1, 3, and 5 days, the cell‐seeded scaffolds were transferred into the sterile 96‐well plate in a one‐to‐one ratio, followed by the addition of 10 μL CCK‐8 solution. The absorbance of the scaffold was measure at 450 nm and all samples were quantified three times.

### 4.3 Chondrogenic differentiation study in vitro and in vivo

#### 4.3.1 Three‐dimensional (3D) scaffold‐drugs‐PBMSCs culture

A suspension of PBMSCs was prepared in a 2% (w/v) alginate solution, with a concentration of 2 × 10^6^ cells/mL. The hardened cross‐linking compound was formed by immersing the gelatin containing PBMSCs in a 102 mM CaCl_2_ solution for 5 min while the gelatin was shaped into a cubic structure (2 × 2 × 2 mm^3^). After 3 weeks of culture in a 12‐well plate containing 2 mL of complete medium, the gelatin complexes were transferred to the plate. Subsequently, histological analysis, mRNA and protein analysis were conducted on the gelatin complexes.

#### 4.3.2 GAIS treatment in osteochondral defect model in mice

In order to establish a subchondral bone model, it is necessary to refer to seminal works in the field.^43^ To summarize, male mice were subjected to anaesthesia using ketamine (40 mg/kg) and xylazine (5 mg/kg). In the presence of the exposed inter‐condyle notch of the distal femur (oriented upwards, specifically in the left lower limb), an osteochondral defect was created using a 21 G needle measuring 1 mm in diameter and 2 mm in height. The defects were implanted with alginate‐gelatin complexes containing PBMSCs and conditioned drugs.

The 3D composites were fabricated in the interim as detailed below. A freshly prepared 2% (w/v) alginate solution was used to contain 2 × 10^−6^ M ICA and 300 ng/mL SDF‐1α. The gelatins exhibited a cubic structure, measuring 2 × 2 × 2 mm^3^. These 2% (w/v) alginate solutions were utilized to resuscitate the PBMSCs in order to obtain a 10^6^/mL cell suspension. In accordance with the methodology outlined in ‘Construction of Alginate‐Coated Gelatin Porous Composites’, a GAIS complex was generated, comprising PBMSCs, ICA and SDF‐1α. Subsequently, GAIS was surgically implanted into the site of the osteochondral defect. The control mice did not undergo any surgical intervention, while the mice in the model groups were exclusively subjected to subchondral bone injury surgery. Mice in the GAIS groups were concurrently administered GAIS treatment and subjected to surgery. Subsequently, the mice were euthanized 12 weeks after the surgery, and their left femur was obtained. The surface cartilage of the distal femur was shaved off with a blade. Subsequently, WB, qPCR and histological assay were utilized to assess and analyse the expression of genes and proteins.

#### 4.3.3 Histological staining and assessment

Traditional histological techniques were utilized to section the cultured 3D complexes and subchondral defect samples using 5 μm paraffin. Hematologic evaluation was conducted utilizing Alcian blue, SO and HE staining. As outlined by the International Cartilage Repair Society (ICRS) II, the degree of subchondral bone repair was evaluated by three readers who were blinded. The histological parameters for ICRS II were modified to specify the matrix integrity (0%, no staining; 100%, full metachromasia) and cartilage surface integrity (0%, abnormal; 100%, normal).^44^

#### 4.3.4 Western blotting

The protein concentrations were determined using a BCA kit (#P0009, Beyotime, Shanghai, China). The protein samples, each containing 30 μg, were subjected to separation through SDS PAGE using a 10% gel. Subsequently, the proteins were transferred onto PVDF (polyvinylidene difluoride) membranes using a Bio‐Rad semi‐dry transfer system (Bio‐Rad Laboratories, Inc., Hercules, CA, USA). Thereafter, the proteins were transferred onto a polyvinylidene difluoride membrane. Following the blockade of the membrane using fetal bovine serum, the membrane was subjected to incubation with primary rabbit antibodies targeting glyceraldehyde 3‐phosphate dehydrogenase (GAPDH; 1:1000; sc‐365062, Santa, USA) and SOX9 (1:1000; sc‐166505, Santa) at a temperature of 4°C overnight. Subsequently, the membranes were subjected to incubation with a secondary antibody (cat. no. ARG65350; 1:3000 dilution; Arigo Biolaboratories Corp., Taiwan, China) that was labelled with horseradish peroxidase. This incubation was carried out for a duration of 1 h at room temperature. Quantity One 4.6.7 software (Bio‐Rad Laboratories, Inc.) was used to obtain the band grey value; GAPDH was taken as the internal reference, and the expression of all proteins were calculated by dividing the grey value of the target protein band by the grey value of the GAPDH band.

#### 4.3.5 Real time quantitative PCR (qPCR)

The mRNA was isolated from the treated PBMSCs and subsequently transcribed into complementary DNA (cDNA) using the PrimeScript RT Master Mix. The qPCR was conducted using the SYBR Premix ExTaq reagent on the qTOWER version 3.0 PCR system. The primers for the target gene are presented in Table 1. The 2^−ΔΔCt^ method was utilized to calculate the expression of all genes, with GAPDH serving as the control.^45^

**TABLE 1:** The primer sequences of gene amplification.

Genes	Forward	Reverse
GAPDH	5′‐AAGTTCAACGGCACAGTCAAGG‐3′	5′‐GACATACTCAGCACCAGCATCAC‐3′
SOX9	5′‐GGCGGAGGAAGTCGGTGAAG‐3′	5′‐AGATGGCGTTAGGAGAGATGTGAG‐3′
COL2A1	5′‐GGAGCAGCAAGAGCAAGGAGAAG‐3′	5′‐GGAGCCCTCAGTGGACAGTAGAC‐3′
ACAN	5′‐CACAGGCAGCACAGACACTTC‐3′	5′‐GGAGTCAAGGTCGCCAGAGG‐3′
MMP13	5′‐TTTGAGAACACGGGGAAGA‐3	5′‐ACTTTGTTGCCAATTCCAGG‐3′

#### 4.3.6 Data collection, statistics and analysis

The data in this study were sorted and analysed using GraphPad Prism (Version 9.0) by one‐way analysis of variance (ANOVA) method. The results are presented as the mean ± standard deviation. The experiments and statistical analyses were conducted with a minimum of three repetitions or more. Statistical significance was attributed to differences that had a *p*‐value less than 0.05.

## AUTHOR CONTRIBUTIONS

**Pengzhen wang:** Conceptualization (equal); funding acquisition (equal); methodology (equal); project administration (equal); resources (equal); software (equal); writing – original draft (equal). **Pingping Zhu:** Conceptualization (equal); data curation (equal); methodology (equal); project administration (equal); resources (equal); writing – original draft (equal). **Wenhui Yin:** Data curation (equal); methodology (equal); project administration (equal); software (equal); supervision (equal); validation (equal). **Jian Wu:** Formal analysis (equal); investigation (equal); supervision (equal); visualization (equal). **Shaoheng Zhang:** Conceptualization (equal); data curation (equal); formal analysis (equal); resources (equal); writing – original draft (equal); writing – review and editing (equal).

## FUNDING INFORMATION

This work was supported by the Medical Science and Technology Research Foundation of Guangdong (A2021335, PW), Traditional Chinese Medicine Bureau of Guangdong Province (20222166, PW), Guangdong Provincial Basic and Applied Basic Regional Joint Fund (2020A1515110009, PZ), Guangzhou Science and Technology Bureau City School (Institute) Enterprise Joint Project (2024A03J0653, PW), Guangzhou Science and Technology Bureau City School (Institute) Enterprise Joint Project (2024A03J0564, JW) and Research Grant of Key Laboratory of Regenerative Medicine, Ministry of Education, Jinan University (ZSYXM202305, PW).

## CONFLICT OF INTEREST STATEMENT

The authors confirm that there are no conflicts of interest.

## Data Availability

The datasets in the present study could be available from the corresponding author on reasonable request.
