# Corrigendum to “ICA/SDF‐1α/PBMSCs Loaded Onto Alginate and Gelatin Cross‐Linked Scaffolds Promote Damaged Cartilage Repair”

**DOI:** 10.1111/jcmm.71417

**Published:** 2026-10-30

P. Wang , P. Zhu , W. Yin , et al., “ICA/SDF‐1α/PBMSCs Loaded Onto Alginate and Gelatin Cross‐Linked Scaffolds Promote Damaged Cartilage Repair,” Journal of Cellular and Molecular Medicine 28, no. 7 (2024): e18236, 10.1111/jcmm.18236.PMC1095515738509746

The original Figure 4A provided was incorrect. The correct Figure 4A is found here. The authors confirmed that these errors do not affect the overall conclusions of the study.

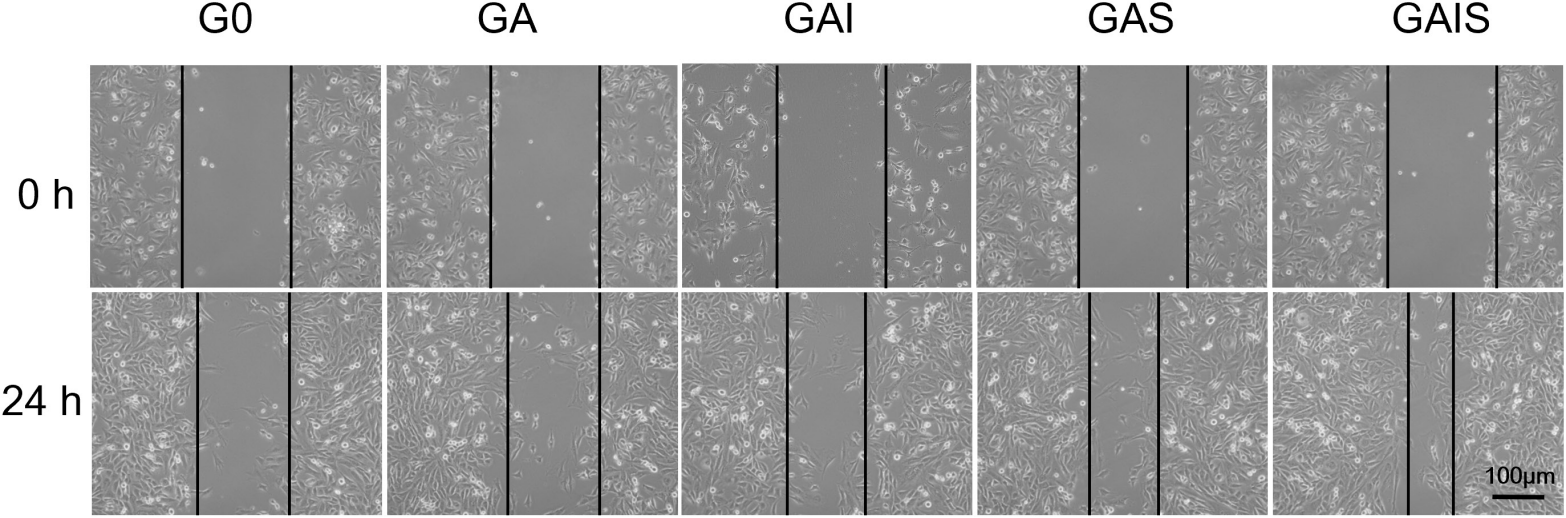

We apologize for this error.

